# Contributions of Spore Secondary Metabolites to UV-C Protection and Virulence Vary in Different Aspergillus fumigatus Strains

**DOI:** 10.1128/mBio.03415-19

**Published:** 2020-02-18

**Authors:** Adriana Blachowicz, Nicholas Raffa, Jin Woo Bok, Tsokyi Choera, Benjamin Knox, Fang Yun Lim, Anna Huttenlocher, Clay C. C. Wang, Kasthuri Venkateswaran, Nancy P. Keller

**Affiliations:** a Biotechnology and Planetary Protection Group, Jet Propulsion Laboratory, California Institute of Technology, Pasadena, California, USA; b Department of Pharmacology and Pharmaceutical Sciences, School of Pharmacy, University of Southern California, Los Angeles, California, USA; c Department of Medical Microbiology and Immunology, University of Wisconsin—Madison, Madison, Wisconsin, USA; d Department of Pediatrics, University of Wisconsin—Madison, Madison, Wisconsin, USA; e Department of Chemistry, Dornsife College of Letters, Arts, and Sciences, University of Southern California, Los Angeles, California, USA; f Department of Bacteriology, University of Wisconsin—Madison, Madison, Wisconsin, USA; Duke University Medical Center

**Keywords:** *Aspergillus fumigatus*, space station, UV-C, melanin, secondary metabolite, spore, virulence determinants

## Abstract

Fungi are versatile organisms which thrive in hostile environments, including the International Space Station (ISS). Several isolates of the human pathogen Aspergillus fumigatus have been found contaminating the ISS, an environment with increased exposure to UV radiation. Secondary metabolites (SMs) in spores, such as melanins, have been shown to protect spores from UV radiation in other fungi. To test the hypothesis that melanin and other known spore SMs provide UV protection to A. fumigatus isolates, we subjected SM spore mutants to UV-C radiation. We found that 1,8-dihydroxynaphthalene (DHN)-melanin mutants of two clinical A. fumigatus strains (Af293 and CEA17) but not an ISS-isolated strain (IF1SW-F4) were more sensitive to UV-C than their respective wild-type (WT) strains. Because DHN-melanin has been shown to shield A. fumigatus from the host immune system, we examined all DHN mutants for virulence in the zebrafish model of invasive aspergillosis. Following recent studies highlighting the pathogenic variability of different A. fumigatus isolates, we found DHN-melanin to be a virulence factor in CEA17 and IF1SW-F4 but not Af293. Three additional spore metabolites were examined in Af293, where fumiquinazoline also showed UV-C-protective properties, but two other spore metabolites, monomethylsulochrin and fumigaclavine, provided no UV-C-protective properties. Virulence tests of these three SM spore mutants indicated a slight increase in virulence of the monomethylsulochrin deletion strain. Taken together, this work suggests differential roles of specific spore metabolites across Aspergillus isolates and by types of environmental stress.

## Abstract (precis)

Fungal spores contain secondary metabolites that can protect them from a multitude of abiotic and biotic stresses. Conidia (asexual spores) of the human pathogen Aspergillus fumigatus synthesize several metabolites, including melanin, which has been reported to be important for virulence in this species and to be protective against UV radiation in other fungi. Here, we investigate the role of melanin in diverse isolates of A. fumigatus and find variability in its ability to protect spores from UV-C radiation or impact virulence in a zebrafish model of invasive aspergillosis in two clinical strains and one ISS strain. Further, we assess the role of other spore metabolites in a clinical strain of A. fumigatus and identify fumiquinazoline as an additional UV-C-protective molecule but not a virulence determinant. The results show differential roles of secondary metabolites in spore protection dependent on the environmental stress and strain of A. fumigatus. As protection from elevated levels of radiation is of paramount importance for future human outer space explorations, the discovery of small molecules with radiation-protective potential may result in developing novel safety measures for astronauts.

## INTRODUCTION

Recent revitalization of the interest in human outer space explorations has brought attention to the question of whether and how microbes, particularly pathogenic microbes, respond and adapt to space conditions (1–4). Being ubiquitous in the environment and isolated from a variety of extreme and built environments, fungi are compelling microorganisms to evaluate such adaptive responses (5–9). Further, fungal presence aboard spacecraft, including the International Space Station (ISS), has been documented (10–12). Of concern is the occurrence of potential human pathogens, such as Aspergillus fumigatus, the causal agent of invasive aspergillosis, in the ISS (1).

UV radiation is known to be enhanced in spacecraft and stations (13). UV may be detrimental to living organisms since it causes DNA damage and induces mutations in both direct and indirect ways by forming pyrimidine dimers and inducing oxidative stress via the generation of reactive oxygen species (ROS), respectively (14, 15). Among three wave bands, UV-A (320 to 400 nm), UV-B (290 to 320 nm), and UV-C (100 to 290 nm), UV-C is known for its germicidal properties and is used as a sanitizing agent (14, 16, 17). In order to protect from damaging UV exposure, fungi have developed various defense mechanisms, including enzymes degrading ROS, DNA repair mechanisms like photoreactivation and nucleotide excision repair, and the production of UV-protective compounds like melanins and other secondary metabolites (SMs) (18).

Fungi produce a diverse variety of SMs that can protect a fungus from harmful environmental factors or from competing microorganisms (19). Several studies have correlated increased conidial melanin content with UV protection in highly irradiated areas, including Chernobyl Power Plant accident sites, the ISS, and “Evolution Canyon” (20–22). Further, albino mutants have been shown to have less fitness in the field, presumably due to the loss of melanin (23). In addition to protecting fungi from UV radiation, fungal melanins are potent virulence factors in both human-pathogenic (e.g., Cryptococcus neoformans [24], Fonsecaea pedrosoi [25], and A. fumigatus [26]) and plant-pathogenic (e.g., Venturia inaequalis [27] and Verticillium dahliae [28]) fungi.

The A. fumigatus conidial melanin, 1,8-dihydroxynaphthalene (DHN)-melanin, thwarts immune cell attack through various mechanisms, including masking of fungal pathogen-associated molecular patterns (PAMPs) and sequestration of signaling molecules such as calcium (29, 30). However, it is unknown if this pigment provides protection to A. fumigatus from UV radiation. The findings that two ISS-isolated strains of A. fumigatus showed increased virulence in a zebrafish model of invasive aspergillosis (IA) (1) and also presented an increase in proteins associated with SM biosynthesis, including trypacidin, Asp-hemolysin, and melanin (3), compared to clinical strains CEA10 and Af293, suggested a possible correlation between conidium-associated SMs, UV-C sensitivity, and virulence.

To address any possible linkage of increased UV-C resistance and virulence mechanisms of pathogenic fungi aboard the ISS, the UV-C sensitivity and pathogenicity of DHN-melanin mutants in three diverse strains of A. fumigatus, including one ISS strain, were evaluated. Because UV is also absorbed by additional aromatic fungal SMs and certain amino acids such as tryptophan and tyrosine (31, 32), any role for three aromatic conidial SMs (trypacidin, fumiquinazoline, and fumigaclavine) for protection from UV-C or impact on virulence in one A. fumigatus strain were also examined. Finally, we examined any role of the DNA repair *ku70* and *ku80* mutants on UV-C sensitivity, as these mutants are frequently used for molecular manipulation of the A. fumigatus genome due to the ease of gene replacement in these backgrounds and the general robust nature of these mutants (33). Due to an existing gap in our understanding of UV-C resistance and virulence mechanisms and to the persistence of fungi aboard the ISS, it was prudent to investigate any possible correlations which may result in developing preventive measures for outer space explorations.

## RESULTS

### Loss of *aku* leads to enhanced UV-C sensitivity but has no effect on secondary metabolite production.

**FIG 1:**
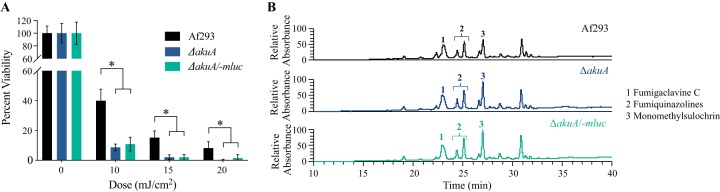
UV-C sensitivity and secondary metabolite profiles of Af293, Δ*akuA* mutant, and Δ*akuA -mluc* mutant strains. (A) Percent viability following exposure to various doses of UV-C radiation of control strains. (B) Secondary metabolite profiles of control strains.

Three control strains, namely, Af293, an Δ*akuA* mutant, and a Δ*akuA -mluc* mutant, were examined to demonstrate the impact of the disruption of *akuA* in UV-C response. This deletion allows for homologous recombination and thus creates a fungus that is easy to genetically manipulate and allows for faster creation of specific mutant strains (33). However, AkuA is involved in DNA repair (34) and would be expected to show enhanced sensitivity to UV treatment. The disruption of *akuA* significantly decreased the survival rate, at 90%, in contrast to 60% decrease in survival of wild-type (WT) Af293 upon UV-C exposure (Fig. 1A); however, there were no differences in UV-C sensitivity between the Δ*akuA* mutant and the Δ*akuA mluc* strain (35), which was also used for gene deletion studies (see Table S1 in the supplemental material). Despite an increase in sensitivity to UV-C, the Δ*akuA* mutant strains still demonstrated a dose response to increasing UV-C and thus were deemed suitable for this study. There were no differences in SM profiles between the WT Af293, Δ*akuA* mutant, and Δ*akuA -mluc* mutant strains (Fig. 1B).

10.1128/mBio.03415-19.5 TABLE S1 Strains used in this study. Download Table S1, DOCX file, 0.1 MB. Copyright © 2020 Blachowicz et al. 2020 Blachowicz et al. This content is distributed under the terms of the Creative Commons Attribution 4.0 International license.

### DHN-melanin is a UV-C protectant in some A. fumigatus isolates.

**FIG 2:**
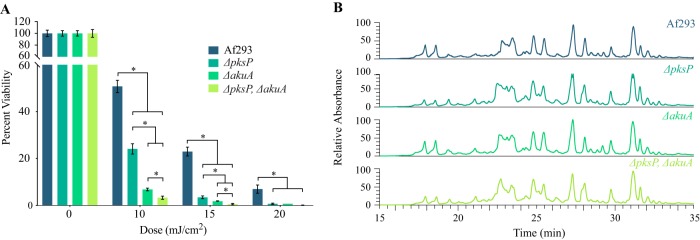
UV-C sensitivity and secondary metabolite profiles of Af293 and Af293 DHN-melanin mutants in the intact and disrupted *akuA* backgrounds. (A) Percent viability following exposure to various doses of UV-C radiation for Af293 and the *ΔpksP*, *ΔakuA*, and *ΔpksP ΔakuA* mutants. (B) Secondary metabolite profiles of Af293 and the *ΔpksP*, *ΔakuA*, and *ΔpksP ΔakuA* mutants. Asterisks (*) indicate statistical significance using Welch’s corrected *t* test (details in Table S2).

Fungal melanins are derived either from an l-dopa biosynthetic pathway (e.g., C. neoformans) or a polyketide biosynthetic pathway (A. fumigatus DHN-melanin) (36). DHN-melanin was tested as a protective molecule from UV-C radiation by deleting *pksP* encoding the polyketide synthase required for DHN-melanin biosynthesis (37) in both WT Af293 and in the Af293 Δ*akuA* background. As shown in Fig. 2A, the loss of *pksP* resulted in strains more sensitive to UV-C regardless of the presence or absence of *akuA*. This increase in sensitivity was seen at all UV-C doses in a comparison of the WT to the Δ*pksP* mutant (∼50% versus ∼25% survival rate at 10 mJ/cm^2^, ∼23% versus ∼3.5% at 15 mJ/cm^2^, and ∼7% versus ∼0.9% at 20 mJ/cm^2^, respectively) and in a comparison of the Δ*akuA* mutant to the Δ*akuA* Δ*pksP* double mutant (∼7% versus ∼3% at 10 mJ/cm^2^, 2% versus 0.5% at 15 mJ/cm^2^, and ∼1% versus 0.1% at 20 mJ/cm^2^, respectively) (Table S2A). Chemical profiling of these strains did not show any differences in the production of other SMs due to the loss of *pksP* (Fig. 2B), but, as expected, both Δ*pksP* and Δ*akuA* Δ*pksP* conidia showed a lack of DHN-melanin pigmentation (Fig. S1).

10.1128/mBio.03415-19.1 FIG S1 WT and DHN-melanin mutant strains of clinical isolates Af293 and CEA17 and ISS isolate IF1SW-F4. Download FIG S1, TIF file, 0.5 MB. Copyright © 2020 Blachowicz et al. 2020 Blachowicz et al. This content is distributed under the terms of the Creative Commons Attribution 4.0 International license.

10.1128/mBio.03415-19.6 TABLE S2 (A) Statistical analysis of UV-C survival of Δ*pksP* mutant strains in disrupted and intact *akuA* backgrounds. (B) Statistical analysis of UV-C survival of CEA17 and Δ*pksP* mutant strains. (C) Statistical analysis of UV-C survival of IF1SW-F4 and Δ*pksP* mutant strains. (D) Statistical analysis of UV-C survival of strains deficient in the production of conidium-associated SMs with intact *akuA*. (E) Statistical analysis of UV-C survival of strains deficient in the production of conidium-associated SMs in an Δ*akuA* background. Download Table S2, XLSX file, 0.1 MB. Copyright © 2020 Blachowicz et al. 2020 Blachowicz et al. This content is distributed under the terms of the Creative Commons Attribution 4.0 International license.

**FIG 3:**
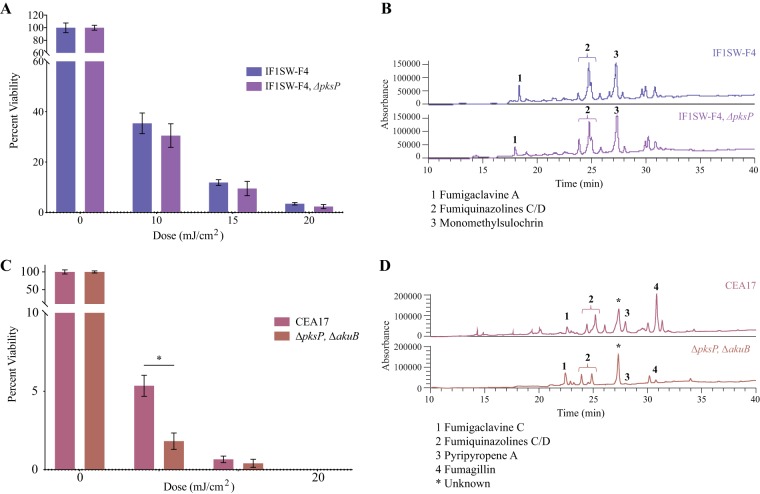
UV-C sensitivity and secondary metabolite profiles of ISS-isolated IF1SW-F4, CEA17, and their DHN-melanin mutants. (A) Percent viability following exposure to various doses of UV-C radiation for IF1SW-F4 and IF1SW-F4 *ΔpksP*. (B) Secondary metabolite profiles of IF1SW-F4 and IF1SW-F4 *ΔpksP*. (C) Percent viability following exposure to various doses of UV-C radiation for CEA17 and CEA17 *ΔpksP*. (D) Secondary metabolite profiles of CEA17 and CEA17 *ΔpksP*. Asterisks (*) indicate statistical significance using Welch’s corrected *t* test (details in Table S2).

Since DHN-melanin was critical in protecting conidia from UV-C radiation in the Af293 background, it was further examined whether this role was conserved in other A. fumigatus isolates, including the ISS-isolated strain IF1SW-F4. Upon exposure to UV-C, it was observed that PksP loss did not significantly diminish the survival of the ISS isolate compared to respective control strain producing DHN-melanin (Fig. 3A and Table S2C). SMs were quantified in these strains and showed no differences in the IF1SW-F4 WT or Δ*pksP* strains (Fig. 3B). Simultaneously, the impact of DHN-melanin on UV-C sensitivity was tested in CEA17 (CEA17 is a CEA10 derivative deleted for AkuB, which forms the functional heterodimer with AkuA [38, 39]). Unlike the IF1SW-F4 Δ*pksP* mutant but similar to the Af293 Δ*pksP* mutants, the CEA17 Δ*pksP* strain showed a significantly decreased survival rate compared to that of the CEA17 control (Fig. 3C and Table S2B), with values similar to those observed in Af293 with the disrupted AkuA background (∼5% versus ∼2% survival rate at 10 mJ/cm^2^ and ∼0.6% versus ∼0.4% at 15 mJ/cm^2^, respectively). Further, the CEA17 Δ*pksP* strain showed alterations in its SM profile, including decreased yields of pyripyropene A and fumagillin compared to the control strain (Fig. 3D, peaks 3 and 4, respectively).

### Fumiquinazoline provides protection from UV radiation.

**FIG 4:**
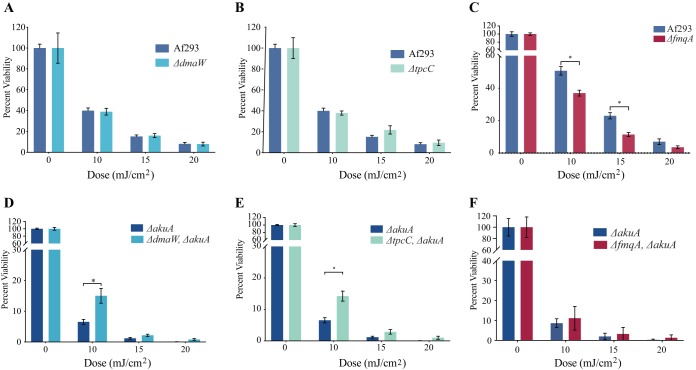
UV-C sensitivity and secondary metabolite profiles of conidium-associated SM mutants of Af293 in backgrounds with disrupted and intact *akuA*. (A to C) Percent viability following exposure to various doses of UV-C radiation for *ΔdmaW* (A), *ΔtpcC* (B), and *ΔfmqA* (C) mutants in Af293 with intact *akuA*. (D to F) Percent viability following exposure to various doses of UV-C radiation for *ΔdmaW* (D), *ΔtpcC* (E), and *ΔfmqA* (F) in Af293 with disrupted *akuA*. Asterisks (*) indicate statistical significance using Welch’s corrected *t* test (details in Table S2).

Aspergillus fumigatus produces other conidial metabolites, including fumigaclavine, trypacidin, and fumiquinazoline (40–42) (Fig. S2). Due to their synthesis in the spore as well as aromatic structure and incorporation of tryptophan in two of these metabolites (fumigaclavine and fumiquinazoline), they were tested to determine if they might also provide protection from UV-C in Af293. Mutants were created for all three metabolites in both WT *akuA* and Δ*akuA* mutant backgrounds (Fig. S3). SM analysis of these deletion mutants confirmed that they did not synthesize the targeted metabolite. Specifically, fumigaclavine C (366.5 g/mol), monomethylsulochrin (346.3 g/mol), and fumiquinazolines C and D (443.5 g/mol) were not detected in extracted ion chromatograms (EICs) of the respective deletion mutants (Fig. S4). Considering that trypacidin is not detectable until 9 to 10 days of growth, monomethylsulochrin (the final intermediate of the trypacidin biosynthetic pathway) was used to confirm the disruption of the trypacidin biosynthetic pathway. In the WT background, the fumiquinazoline mutant (Δ*fmqA*) was more sensitive to UV-C (35%) (Fig. 4C and Table S2D), but the loss of either fumigaclavine (Δ*dmaW* mutant) or trypacidin (Δ*tpcC* mutant) pathway metabolites had no impact on spore survival (Fig. 4A and B and Table S2D). However, examination of these same SM mutations in the Δ*akuA* background gave different results, where strains deficient in fumigaclavine A (Δ*akuA* Δ*dmaW* mutant) and trypacidin (Δ*akuA* Δ*tpcC* mutant) were slightly more resistant to UV-C than was the Δ*akuA* control after exposure to 10 mJ/cm^2^ (*P* = 0.0043 and 0.0006, respectively) (Fig. 4D and E and Table S2E) and the fumiquinazoline null strain (Δ*akuA* Δ*fmqA* mutant) showed no significant change in UV-C resistance compared to Δ*akuA* (Fig. 4F and Table S2D compared to 2E). These findings suggested that *akuA* loss alters the UV-C-protective properties of some conidial pigments and may allow for the identification of mutants that can enhance UV-C resistance.

10.1128/mBio.03415-19.2 FIG S2 Chemical structures of conidium-associated secondary metabolites. Download FIG S2, PDF file, 0.9 MB. Copyright © 2020 Blachowicz et al. 2020 Blachowicz et al. This content is distributed under the terms of the Creative Commons Attribution 4.0 International license.

10.1128/mBio.03415-19.3 FIG S3 Southern blot confirmation of the secondary metabolite mutants. Download FIG S3, PDF file, 2.5 MB. Copyright © 2020 Blachowicz et al. 2020 Blachowicz et al. This content is distributed under the terms of the Creative Commons Attribution 4.0 International license.

10.1128/mBio.03415-19.4 FIG S4 Secondary metabolite profiles of conidium-associated secondary metabolite mutants. Download FIG S4, PDF file, 1.5 MB. Copyright © 2020 Blachowicz et al. 2020 Blachowicz et al. This content is distributed under the terms of the Creative Commons Attribution 4.0 International license.

### DHN-melanin role in virulence in three A. fumigatus isolates does not correlate with its role in UV-C protection.

**FIG 5:**
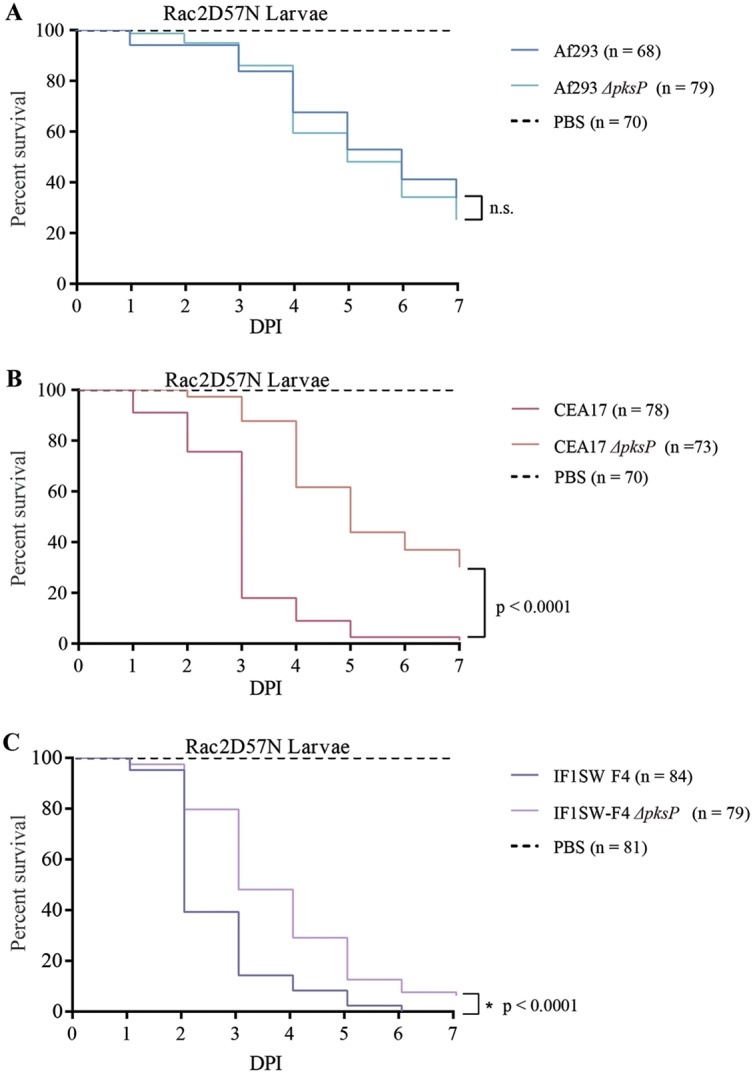
Virulence of DHN-melanin mutants of three Aspergillus fumigatus strains in a zebrafish model of invasive aspergillosis. (A) Percent survival of zebrafish upon infection with Af293 and the *ΔpksP* mutant. (B) Percent survival of zebrafish upon infection with CEA17 and the *ΔpksP* mutant. (C) Percent survival of zebrafish upon infection with IF1SW-F4 and *ΔpksP* mutant. *P* values were generated by Cox proportional hazards regression analysis. DPI, days postinfection.

Considering the differential role of DHN-melanin in shielding the three strains from UV-C damage (Fig. 2 and 3), the known role of DHN-melanin in the virulence of some A. fumigatus strains (35), and differences in virulence between Af293 and CEA10 strains (43–46), we thought it important to assess the virulence of the three Δ*pksP* mutants. *pksP* deletion mutants were examined for changes in virulence relative to their respective WT controls using an established neutrophil-defective (*rac2D57N*) zebrafish model of invasive aspergillosis (46, 47). Whereas both the CEA17 and IF1SW-F4 Δ*pksP* mutant strains were significantly decreased in virulence, the Af293 Δ*pksP* mutant was not (Fig. 5).

**FIG 6:**
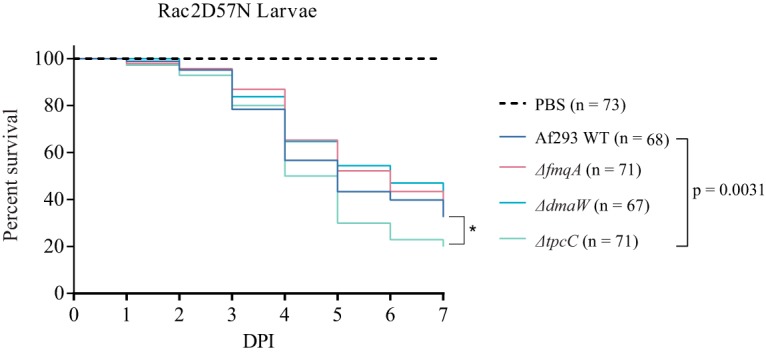
Virulence of three conidial SM mutants of Aspergillus fumigatus in a zebrafish model of invasive aspergillosis. Percent survival of zebrafish upon infection with Af293 WT and the *ΔdmaW*, *ΔtpcC*, and *ΔfmqA* mutants. *P* values were generated by Cox proportional hazards regression analysis.

We also assessed the virulence of the Δ*tpcC*, Δ*fmqA*, and Δ*dmaW* mutants in the same zebrafish model. The loss of either *fmqA* or *dmaW* showed no difference in zebrafish deaths in comparison to the Af293 WT; however, the Δ*tpcC* mutant had a 10% increase in zebrafish deaths (Fig. 6). Together, our results suggest that the involvement of spore SMs in virulence is strain dependent and that their protective roles in fungal biology vary with the type of environmental stress.

## DISCUSSION

While the radiation-protective properties of melanin in fungi have been previously documented (21, 22, 48, 49), little is known about the UV-C-protective features of other SMs or of DHN-melanin in A. fumigatus. Since A. fumigatus strains have been isolated from space station environments characterized by high UV-C levels (12) and increased virulence (1), it was prudent to investigate the UV-C properties of conidial SMs and their plausible correlation with pathogenicity. Here, we found that DHN-melanin protects some but not all A. fumigatus isolates from UV-C radiation and that fumiquinazolines are UV-C protectants in WT Af293.

This study demonstrated that *akuA*, which plays fundamental role in the nonhomologous end-joining (NHEJ) pathway, a preferred DNA repair pathway of DNA double-strand breaks (DSBs) in fungi (50–52), is critical in protection from UV-C. The disruption of *akuA* gene, which increases the transformation success rate via homologous recombination (HR), led to a ca. 90% decrease in survival upon exposure to the lowest UV-C dose, 10 mJ/cm^2^, while the Af293 WT showed a decrease of 60% for the same dose (Fig. 1A). Such discrepancy in the survival rates between the Δ*akuA* mutant and the WT was of no surprise, as previous reports showed that UV-C exposure significantly induces DSB incidence in exposed organisms and cells (53, 54). Further, no significant differences were observed in the SM profiles of the tested control strains (Fig. 1B), suggesting that *akuA* gene disruption was the main cause of the observed drop in strain survival upon UV-C exposure. However, we found that using an *akuA* null background could mask the ability to identify UV-C-protective SMs, possibly dependent on the relative contributions of each metabolite. For instance, the deletion of *pksP* in Af293 resulted in increased sensitivity to UV-C regardless of the *akuA* background (Fig. 2), but the protective property of fumiquinazoline was masked in the Δ*akuA* background (Fig. 4C versus F).

Unexpectedly, during the study, it was noticed that the UV-C-protective property of DHN-melanin was strain dependent. Previous studies with other fungi have demonstrated the importance of DHN-melanin in UV protection (21, 22, 48), but an examination of melanin in different isolates within a species has not been reported, to our knowledge. Af293, CEA10, and CEA17 are commonly studied clinical strains of A. fumigatus (33, 45, 55) and in IF1SW-F4, a strain previously reported as being isolated from the ISS (1). Further, DHN-melanin-deficient strain CEA17, but not strain IF1SW-F4, was more sensitive to UV-C (Fig. 3). These data suggest that there might be other metabolites which are important for tolerating the UV-C stress in IF1SW-F4. We also note that different A. fumigatus strains synthesize different SMs and different amounts of each SM which could contribute to differences in UV-C sensitivity (Fig. 3) (1, 42). Such UV-C-protective compounds are of paramount importance during future outer space explorations, as they may be implemented to protect other organisms, including humans and plants (56).

Considering the differential role of DHN-melanin in UV protection coupled with the already-known differences in Af293, CEA10, and CEA17 virulence (43–46, 57, 58), we speculated that PksP might also play a differential role in virulence dependent on A. fumigatus strains. Using the neutrophil-deficient zebrafish model of invasive aspergillosis (46), we found this to be true but not in the manner we had anticipated. We had hypothesized that there might be a correlation between the importance of DHN-melanin in UV-C protection (where PksP protected Af293 and CEA17; Fig. 2 and 3) and virulence, but instead, we found that the CEA17 and IF1SW-F4 Δ*pksP* mutants (but not the Af293 mutant) showed a loss in virulence as measured by zebrafish mortality (Fig. 5). CEA17 showed the most significant attenuation of virulence of the two isolates. Different strains of A. fumigatus, including CEA10 (and derivatives such as CEA17) and Af293, have previously been shown to vary in virulence (in human cells [55], Drosophila melanogaster [43], murine [44, 59] and zebrafish [1, 40] models). DHN-melanin is an important cell wall component that impacts cell wall morphology and assembly (60) and has been shown to be involved in the protection of conidia from the host immune cells in A. fumigatus ATCC 46645 (61). The results from both the UV-C sensitivity tests together with the virulence assessment suggest that the role of DHN-melanin in conidial protection varies with the type of environmental stress.

This work also presented the findings that other conidial SMs, in this case, fumiquinazoline, can protect from UV-C radiation. Fumiquinazoline C is composed of three residues, including the aromatic amino acid tryptophan and its precursor anthranilic acid (62). We hypothesize that this metabolite may contribute to UV production, possibly through tryptophan- and anthranilic acid-absorbing properties, and that its protective properties were masked in the highly UV-sensitive Δ*akuA* background (Fig. 4). We found that the loss of fumigaclavine or trypacidin intermediates had no impact on UV-C protection in an AkuA WT background, and these strains were actually more resistant to UV-C in the *akuA* background than control strains (Fig. 4). The increased resistance might possibly be explained by findings where the loss of one SM often results in increased synthesis of another SM (63), possibly one with some UV-C protection in this case. We had thought that trypacidin loss would result in increased sensitivity to UV-C, as several of its precursors (e.g., emodin and questin) are pigmented anthraquinones that have been shown to protect Xanthoria elegans and Cetraria islandica against UV (42, 64). Possibly, there are not enough anthraquinones synthesized in Af293 to have a measurable effect on UV sensitivity. We further assessed the impact of the loss of these SMs on virulence in the zebrafish model and found no correlation to sensitivity to UV-C (Fig. 4 and 6). These results parallel our observations on DHN-melanin loss, namely, that each SM may have differential role dependent on environmental stress.

This study is the first comprehensive report to show the impact of conidium-associated SMs of A. fumigatus on UV-C protection and virulence. The results demonstrated strain-dependent involvement of DHN-melanin in both UV-C protection and virulence, highlighting that the role of fungal SMs cannot be generalized across a species. We also identify fumiquinazoline as playing a role in UV-C protection in strain Af293. In-depth analyses with regard to A. fumigatus virulence factors and UV-C resistance are important especially in isolated environments such as the space station. IF1SW-F4, isolated from the ISS and shown to be more virulent than both Af293 and CEA10 (1), but possibly, the molecules and properties leading to increased virulence are different from those of Af293 or CEA10. Such understanding may help develop and promote safety precautions for astronauts during long-term space explorations.

## MATERIALS AND METHODS

### Fungal strains and growth conditions.

The strains used in this study are listed in Table S1. The generated mutants were stored in 30% (vol/vol) glycerol in 0.01% (vol/vol) Tween 80 at −80°C. Strains were activated and grown at 37°C on glucose minimal medium (GMM; 6 g/liter NaNO_3_, 0.52 g/liter KCl, 0.52 g/liter MgSO_4_·7H_2_O, 1.52 g/liter KH_2_PO_4_, 10 g/liter d-glucose, 15 g/liter agar supplemented with 1 ml/liter trace elements) for 3 days to collect and enumerate conidia in 0.01% (vol/vol) Tween 80. For UV-C exposure and secondary metabolite (SM) analysis, approximately 10^2^ conidia per plate (diameter, 10 cm) were suspended in 5 ml of GMM agar containing half of the agar concentration (top agar) and grown at 37°C for 7 days. For assessment of virulence in larval zebrafish, plates were inoculated with 1 × 10^6^ conidia of the appropriate fungal strain and incubated at 37°C for 3 days. Conidia were harvested in 0.01% (vol/vol) Tween 80 using a cell spreader and filtered through Miracloth. Conidial stocks for microinjection were resuspended in Dulbecco’s phosphate-buffered saline (PBS) at a concentration of 1.5 × 10^8^ conidia/ml.

### Construction of mutants.

To create the Δ*pksP* mutant in IF1SW-F4, the CRISPR-Cas9 system developed by the Mortensen lab was used (65). In brief, the IF1SW-F4 was tested for antibiotic sensitivity against hygromycin (hyg) and phleomycin (ble) to determine the selection marker and associated plasmid for targeting *pksP*. The strain showed sensitivity to 80 μg/ml hyg; hence, the plasmid pFC332 containing the hyg cassette was used for fungal transformation. Further, the 20-bp sequence 5′-ATC GCC AGC AAC GCC ACG CA-3′, a protospacer targeting *pksP*, was selected within the *pksP* gene and introduced to the pFC332 plasmid using the custom primers Fw1 (5′-TAG CTG TTT CCG CTG A-3′), Rev1 (5′-TGC GTG GCG TTG CTG GCG ATG ACG AGC TTA CTC GTT TCG TCC TCA CGG ACT CAT CAG ATC GCC CGG TGA TGT CTG CTC AAG-3′), Fw2 (5′-TCG TCA TCG CCA GCA ACG CCA CGC AGT TTT AGA GCT AGA AAT AGC AAG TTA A-3′), and Rev2 (5′-ATT CTG CTG TCT CGG CTG-3′). Upon this step, plasmid was propagated into Escherichia coli DH5α, purified using the QIAprep Spin miniprep kit (Qiagen, Hilden, Germany), and used for transformation. Correct transformants were selected based on the loss of the pigment and used for downstream analyses. For gene deletion mutants, construction of the gene deletion cassette, production of protoplasts, and subsequent polyethylene glycol (PEG) transformation were performed as described previously (66). Briefly, primers (Table S3) were designed containing a 20- to 30-bp overlap were used to amplify 1,000-bp homologous regions of the gene of interest using SeqBuilder (DNAStar, Madison, WI).

10.1128/mBio.03415-19.7 TABLE S3 Primers used in this study. Download Table S3, PDF file, 0.9 MB. Copyright © 2020 Blachowicz et al. 2020 Blachowicz et al. This content is distributed under the terms of the Creative Commons Attribution 4.0 International license.

These flanks were fused to a selectable marker containing either the A. fumigatus *pyrG* or Aspergillus parasiticus *pyrG* gene, amplified from the pKJA12 or pJW24 plasmids, respectively, and the resulting cassette was then transformed into the parent strain via PEG transformation (45). Strains were transformed using either Af293.1, providing a background with an intact *akuA*, or TFYL 80.1 for the Δ*akuA* background. Transformants were screened by selecting transformants that were able to grow in the absence of the appropriate supplements. Loss of the gene of interest and proper integration of the deletion construct were confirmed via PCR and Southern blot analysis (Fig. S3).

### UV-C exposure and survival evaluation.

To evaluate the UV-C sensitivity of studied mutants, approximately 10^2^ conidia/plate were exposed to various UV-C doses. Conidia were suspended in top GMM cooled to 55°C, and 5 ml of this suspension was added to plates with 20 ml of GMM (100% of the agar). Triplicates of each strain were exposed to 10-, 15-, and 20-mJ/cm^2^ doses of UV-C using a Hoefer UVC 500 crosslinker (Amersham Biosciences, Little Chalfont, UK). Upon exposure, treated and untreated (control plates) were incubated at 37°C. After 7 days, CFU were enumerated, and the percent survival was calculated using the following formula: no. of CFU exposed to any given dose (*N*)/CFU in control plate (*N*_0_) × 100. The results from three biological replicates were pooled and used for statistical analysis with GraphPad Prism 8 using Welch’s corrected *t* test.

### Secondary metabolite extraction and analysis.

To examine the production of SMs of interest, agar plugs in triplicate were collected from unexposed plates. Plugs were extracted with 3 ml of methanol (MeOH) and 1:1 MeOH-DCM each, followed by 1 h of sonication. Crude extracts were evaporated *in vacuo* and extracted with 3 ml of ethyl acetate (EtOAc). The EtOAc layer was evaporated *in vacuo* to yield a residue suspended in 1 ml of 20% dimethyl sulfoxide (DMSO)-MeOH, and 10 μl was examined by high-performance liquid chromatography–photodiode array detection–mass spectrometry (HPLC-DAD-MS) analysis. HPLC-MS was carried out using a Thermo Finnigan LCQ Advantage ion trap mass spectrometer with a reverse-phase (RP) C_18_ column (Alltech Prevail C_18_, 3 mm, 2.1 by 100 mm) at a flow rate 125 μl/min. The solvent gradient for LC-MS was 95% methyl cyanide (MeCN)-H_2_O (solvent B) in 5% MeCN-H_2_O (solvent A), both containing 0.05% formic acid, as follows: 0% solvent B from 0 to 5 min, 0% to 100% solvent B from 5 min to 35 min, 100% solvent B from 35 to 40 min, 100% to 0% solvent B from 40 to 45 min, and reequilibration with 0% solvent B from 45 to 50 min.

### Zebrafish care and maintenance.

Adult zebrafish were reared as described previously (46). Briefly, adults were maintained on a dedicated aquatic system and subjected to a light/dark cycle of 14 h/10 h and fed twice a day. After spawning, embryos were collected in E3 buffer supplemented with methylene blue (MB) to inhibit fungal growth and incubated at 28.5°C. At 24 h postfertilization, embryos were manually decoryonated and transferred to E3 buffer lacking MB. Prior to microinjection, larvae were anesthetized in E3 buffer containing 0.2 mg/ml Tricaine (ethyl 3-aminobenzoate; Sigma-Aldrich). Larval zebrafish procedures and adult handling were performed in compliance with NIH guidelines and approved by the University of Wisconsin—Madison Institutional Animal Care and Use Committee.

### Larval zebrafish virulence assay.

Virulence assays were performed using the larval zebrafish model of invasive aspergillosis, as described previously (46), with slight modifications. Neutrophil-defective larvae were obtained genetically through the use of transgenic *mpx:mCherry-2A-rac2D57N* larvae (47). Prior to infection, larvae were screened and selected for mCherry expression in neutrophils to identify individuals harboring the dominant negative allele. Briefly, hindbrain ventricle infections were performed at 48 h postfertilization using conidial stocks that were mixed 2:1 with 1% phenol red (used to visualize injection success) to a final concentration of 1 × 10^8^ conidia/ml stock. Larvae were anesthetized in E3 lacking MB and supplemented with 0.2 mg/ml Tricaine prior to microinjection of 3 nl conidia stock or PBS vehicle control into the hindbrain ventricle through the otic vesicle. Following microinjection, larvae were rinsed several times to remove the anesthetic and transferred to individual wells of a 96-well plate in ∼200 μl E3 lacking MB. The survival of individual larvae was scored daily using a loss of heartbeat as a readout of mortality. Survival analysis for larval zebrafish infection experiments was performed as previously described (46) by pooling experimental replicates and generating *P* values by the Cox proportional hazards regression analysis.
