# Supplementary material for: Contributions of Spore Secondary Metabolites to UV-C Protection and Virulence Vary in Different Aspergillus fumigatus Strains
Source: mBio. 2020 Feb 18;11(1):e03415-19. doi: 10.1128/mBio.03415-19 (PMC7029147; doi:10.1128/mBio.03415-19)
Supplement: TABLE S1 [file mBio.03415-19-st001.docx]

**Supplementary Table S1. Strains used in this study.**

| **Parental Strain** | **Strain ID** | **Strain Name** | **Genotype** | **Publication** |
| --- | --- | --- | --- | --- |
| Af293 | Af293.1 | ∆*pyrG* | *pyrG1* | Xue 2004 (1) |
|  | TFYL 80.1 | ∆*pyrG;*∆*akuA* | *fumiargB; ∆akuA::mluc; pyrG1; argB1* | Lim 2018 (2) |
|  | TFYL81.5 | ∆*akuA* | *fumipyrG; fumiargB; ∆akuA::mluc; pyrG1; argB1* | Throckmorton 2016 (3) |
|  | TBK4.1 | Δ*aku/-mluc** | *fumiargB; ΔakuA::parapyrG; pyrG1; argB1* | Rosowski 2018 (4) |
|  | TTC32.1 | *∆akuA;* ∆*dmaW* | *fumiargB; ∆dmaW::parapyrG; ∆akuA; pyrG1; argB1* | This study |
|  | TJW200.1 | *∆akuA;* Δ*fmqA* | *fumiargB; ∆fmqA::parapyrG; ∆akuA::mluc; pyrG1; argB1* | This study |
|  | TJW184.1 | *∆akuA;* Δ*pksP* | *fumiargB; ΔpksP::parapyrG; ∆akuA; pyrG1* | This study |
|  | TFYL76 | *∆akuA;* *∆tpcC* | *fumipyrG; ∆tpcC::fumiargB; ∆akuA::mluc; pyrG1; argB1* | Throckmorton 2016 (3) |
|  | TTC31.29 | Δ*dmaW* | *ΔdmaW::parapyrG; pyrG1* | This study |
|  | TFYL10.1 | Δ*fmqA* | *∆fmqA::parapyrG; pyrG1* | Lim 2014 (5) |
|  | TJW196.1 | Δ*pksP* | *∆pksP::parapyrG, pyrG1* | This study |
|  | TFYL14.1 | Δ*tpcC* | *ΔtpcC::parapyrG; pyrG1* | This study |
| CEA10 | CEA17 | *∆akuB* | *∆akuB::pyrG; pyrG1* | da Silva Ferreira 2006 (6) |
|  | CEA17 | *∆akuB;*∆*pyrG* | *∆akuB; pyrG1* | D’Enfert 1996 (7) |
|  | TJW184.23 | Δ*pksP; ∆akuB* | Δ*pksP*::*parapyrG*, *∆akuB; pyrG1* | This study |
| IF1SW-F4 | IF1SW-F4-1 | Δ*pksP* | CRISPR-Cas9 disruption | This study |

^*^ *mluc* encodes a luciferase used to monitor pathogen presence in host tissue

1. Xue T, Nguyen CK, Romans A, Kontoyiannis DP, May GS. Isogenic auxotrophic mutant strains in the *Aspergillus fumigatus* genome reference strain AF293. Arch Microbiol. 2004 Nov;182(5):346–53.

2. Lim FY, Won TH, Raffa N, Baccile JA, Wisecaver J, Rokas A, et al. Fungal Isocyanide Synthases and Xanthocillin Biosynthesis in *Aspergillus fumigatus*. mBio [Internet]. 2018 Jul 5 [cited 2019 Dec 12];9(3). Available from: https://mbio.asm.org/content/9/3/e00785-18

3. Throckmorton K, Lim FY, Kontoyiannis DP, Zheng W, Keller NP. Redundant synthesis of a conidial polyketide by two distinct secondary metabolite clusters in *Aspergillus fumigatus*. Environ Microbiol. 2016 Jan;18(1):246–59.

4. Rosowski EE, Raffa N, Knox BP, Golenberg N, Keller NP, Huttenlocher A. Macrophages inhibit *Aspergillus fumigatus* germination and neutrophil-mediated fungal killing. PLOS Pathog. 2018 Aug 2;14(8):e1007229.

5. Lim FY, Ames B, Walsh C, Keller N. Coordination between BrlA regulation and secretion of the oxidoreductase FmqD directs selective accumulation of fumiquinazoline C to conidial tissues in *Aspergillus fumigatus*. Cell Microbiol. 2014 Aug;16(8):1267–83.

6. da Silva Ferreira ME, Kress MRVZ, Savoldi M, Goldman MHS, Härtl A, Heinekamp T, et al. The akuB (KU80) mutant deficient for nonhomologous end joining is a powerful tool for analyzing pathogenicity in *Aspergillus fumigatus*. Eukaryot Cell. 2006 Jan;5(1):207–11.

7. d’Enfert C. Selection of multiple disruption events in *Aspergillus fumigatus* using the orotidine-5’-decarboxylase gene, pyrG, as a unique transformation marker. Curr Genet. 1996 Jun;30(1):76–82.
