# Supplementary material for: Contributions of Spore Secondary Metabolites to UV-C Protection and Virulence Vary in Different Aspergillus fumigatus Strains
Source: mBio. 2020 Feb 18;11(1):e03415-19. doi: 10.1128/mBio.03415-19 (PMC7029147; doi:10.1128/mBio.03415-19)
Supplement: FIG S2 [file mBio.03415-19-sf002.pdf]

A

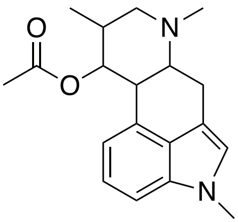

Fumigaclavine C

B

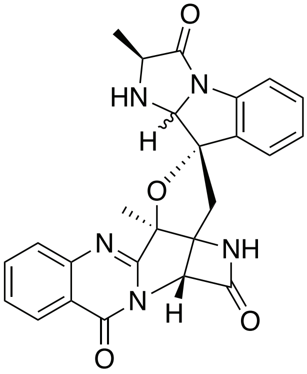

Fumiquinazoline C

C

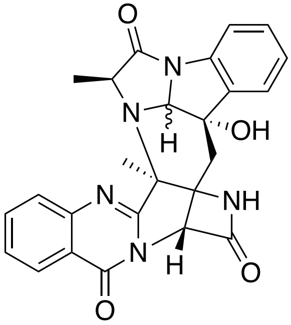

Fumiquinazoline D

D

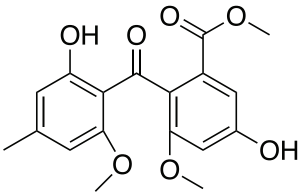

Monomethylsulochrin

E

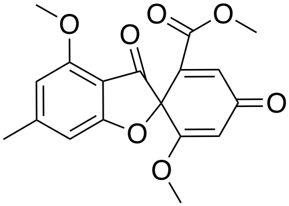

Trypacidin

F

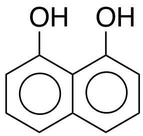

1,8-dihydroxynaphthalene
(DHN-melanin precursor)

Supplementary Figure S2. Chemical structures of conidia associated secondary metabolites.
