# Supplementary material for: Contributions of Spore Secondary Metabolites to UV-C Protection and Virulence Vary in Different Aspergillus fumigatus Strains
Source: mBio. 2020 Feb 18;11(1):e03415-19. doi: 10.1128/mBio.03415-19 (PMC7029147; doi:10.1128/mBio.03415-19)
Supplement: FIG S3 [file mBio.03415-19-sf003.pdf]

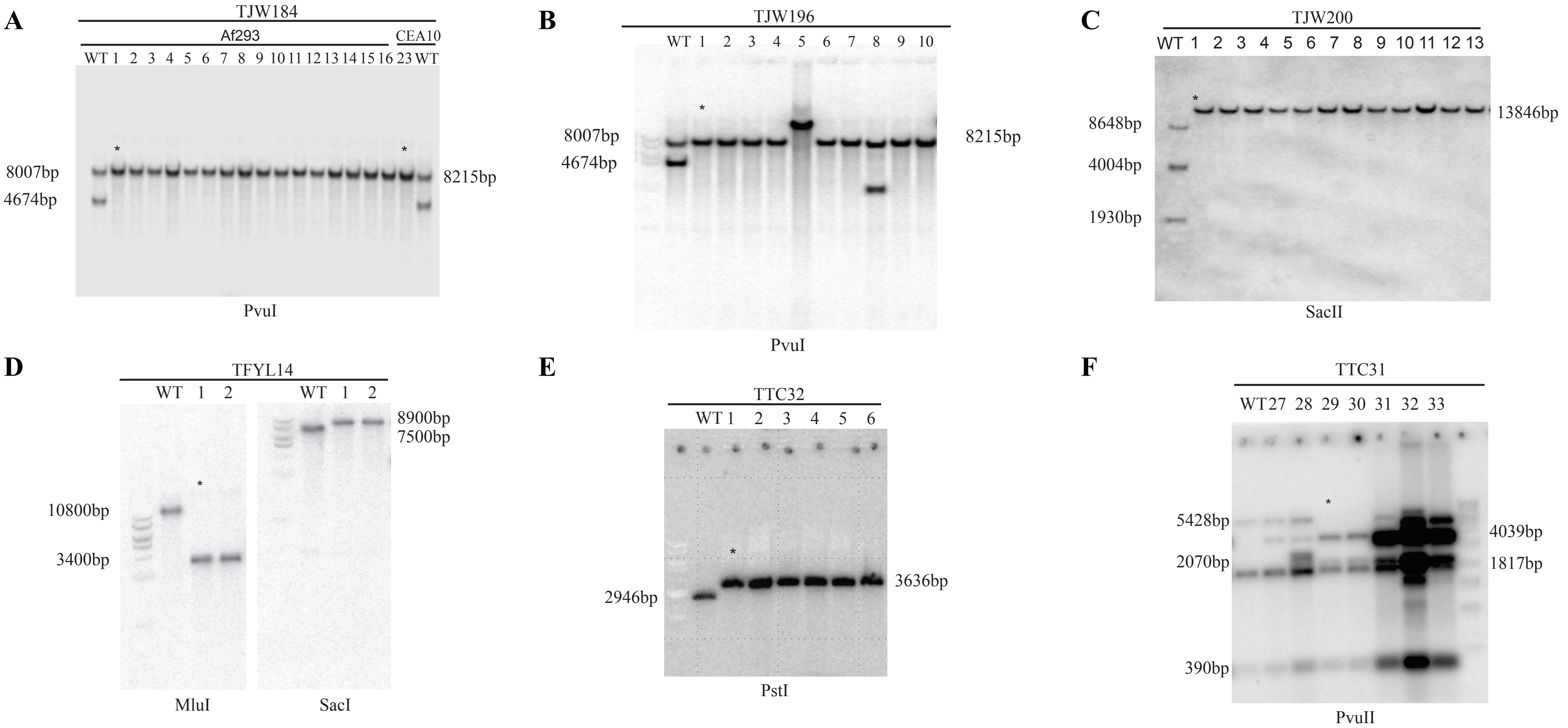

Supplementary Figure S3. Southern confirmation of the secondary metabolite mutants A) *Aspergillus fumigatus* Δ*pksP* southern confirmation in the ku70 deletion background. Genomic DNA was digested by PvuI. Expected band size was wild type (8007 and 4674bp) and ΔpksP (8215bp). TJW184.1 and TJW184.23 were chosen for the subsequent experiments. TFYL80.1 was a parent for TJW184.1 to 16 and CEA17Δ*akuB*; pyrG1 was a parent for TJW184.23. **B)** *Aspergillus fumigatus* Δ*pksP* southern confirmation in wild type ku70 background. Genomic DNA was digested by PvuI. Expected band size was wild type (8007 and 4674bp) and Δ*pksP* (8215bp). CC)TJW196.1, 2, 3, 4, 6, 7, 9, and 10 were correct. TJW196.1 was chosen for the subsequent experiments. Af293.1 was a parent for the deletion. **C)** *Aspergillus fumigatus* Δ*fmqA* southern confirmation in the ku70 deletion background. Genomic DNA was digested by SacII. Expected band size was wild type (8648, 4004 and 1930bp) and Δ*fmqA* (13846bp). All of transformants were correct. TJW200.1 was chosen for the subsequent experiments. TFYL80.1 was a parent for the deletion. **D)** *Aspergillus fumigatus* Δ*tpcC* southern confirmation in the wild type ku70 background. Genomic DNA was digested by SacI and MluI. Expected band size from the MluI digestion was wild type (10800bp) and Δ*fmqA* (3400bp). Expected band size from the SacI mutant was wild type (7500bp) and Δ*fmqA* (8900bp). All of transfor-mants were correct. TFYL14.1 was chosen for the subsequent experiments. Af293.1 was a parent for the deletion. **E)** *Aspergillus fumigatus* Δ*dmaW* southern confirma-tion in deletion ku70 background. Genomic DNA was digested by PstI. Expected band size was wild type (2946bp) and Δ*dmaW* (3636bp). TTC32.1, 2, 3, 4, 5, and 6 were correct. TTC32.1 was chosen for the subsequent experiments. TBK 4.1 was a parent for the deletion. **F)** *Aspergillus fumigatus* Δ*dmaW* southern confirmation in wild type ku70 background. Genomic DNA was digested by PvuII. Expected band size was WT (390bp, 1817bp, and 5428bp) and Δ*dmaW* (390bp, 1817bp, 2070bp, and 4039bp). TTC31.29 and 30 were correct. TTC31.29 was chosen for the subsequent experiments. Af293.1 was a parent for the deletion.
