# Supplementary material for: Contributions of Spore Secondary Metabolites to UV-C Protection and Virulence Vary in Different Aspergillus fumigatus Strains
Source: mBio. 2020 Feb 18;11(1):e03415-19. doi: 10.1128/mBio.03415-19 (PMC7029147; doi:10.1128/mBio.03415-19)
Supplement: FIG S4 [file mBio.03415-19-sf004.pdf]

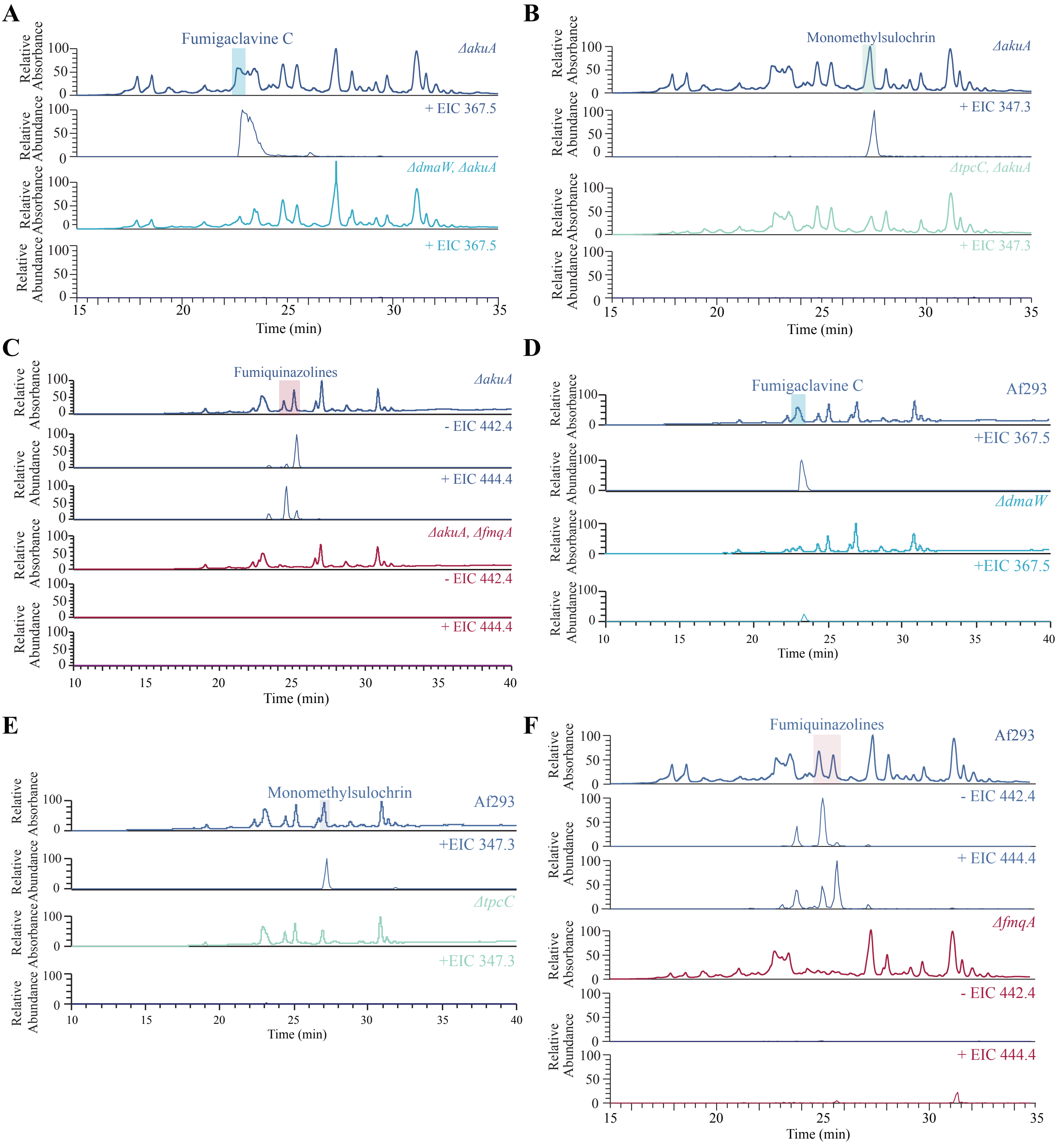

Supplementary Figure S4. Secondary metabolite profiles of conidia associated secondary metabolite mutants.

A, B, C) Secondary metabolite profiles of mutants in Δ*akuA* background Δ*dmaW*, Δ*tpcC* and Δ*fmqA*, Δ*akuA*, respectively.

D, E, F) Secondary metabolite profiles of mutants in *akuA* intact background Δ*dmaW*, Δ*tpcC* and Δ*fmqA*, Δ*akuA*, respectively.
