# Supplementary material for: Contributions of Spore Secondary Metabolites to UV-C Protection and Virulence Vary in Different Aspergillus fumigatus Strains
Source: mBio. 2020 Feb 18;11(1):e03415-19. doi: 10.1128/mBio.03415-19 (PMC7029147; doi:10.1128/mBio.03415-19)
Supplement: TABLE S3 [file mBio.03415-19-st003.pdf]

Supplemental Table S3.
Primers used in this study

	Name	Sequence 5′ - 3′
1	pksPF5	TGTCGATCCTGTGGCATTTGGG
2	pksPR5	CGATATCAAGCTATCGATACCTCGACTCGGCGAGTGGTTTGCGCGGCCGTGACTGC
3	ParapyrGF	GAGTCGAGGTATCGATAGCTTG
4	ParapyrGR	ATTCGACAATCGGAGAGGCTGC
5	pksPF3	GTCGCTGCAGCCTCTCCGATTGTCGAATGTTCCTAGGTTTGGGGGTGGAGTTGTGC
6	pksPR3	TTACTTGGACAGAGGAGAGCGC
7	fmqAF	TGCAGAGACCGAAGAAGCAAGG
8	fmqAR	TCCATCGACTGGTCTTGGATGG
9	14560-5'FOR	TCTGGCCTCCTCTAACCTCA
10	14560-5'REV	CTTATCGATAAGCTGTCAAACATGGTCTATATGCTGGCCCTCCA
11	14560-3'FOR	GTTGATAGCACACCCTCGGAATAGTCTGCTGTCTTCGATTTTGTCG
12	14560-3'REV	GCCCAGGTTCAGGATGTAAA
13	TC- DAfu2g18040 F1	TGAAGGGAAGGGTGGTAGATTCG
14	TC- DAfu2g18040 R1	TATCAAGCTATCGATACCTCGACTCGAGAGTCATCTTGGATAAGGGCATATGC
15	TC- DAfu2g18040 F2	TCGCTGCAGCCTCTCCGATTGTCGAATTGAACAGCTCGGGACTTACTTCC
16	TC- DAfu2g18040 R2	TCGAGATCGGAGTTGTCTTTGG
17	pyrG_prom_F	CGTAATACGACTCACTATAGGG
18	pyrG_term_R	ATTCGACAATCGGAGAGGCTGC
19	TC-DAfu2g18040 F3	ATCCCTTTTGTCCAGAGCAACG
20	TC- DAfu2g18040 R3	TGACCCATATACCCACTCAAGG
21	TC-DAfu2g18040 F4	AGAAGATGTCTGGGTTCTGGTGG
22	TC-DAfu2g18040 R4	ACGGGCAGCACCTAATATACC
